# Measuring force of infection and vaccine effects on transmission stages in clinical trials of experimental malaria vaccines

**DOI:** 10.1186/1475-2875-11-S1-O50

**Published:** 2012-10-15

**Authors:** Ingrid Felger, Blaise Genton, Hans-Peter Beck, Tom Smith

**Affiliations:** 1 Swiss Tropical and Public Health Institute, 4002 Basel, Switzerland

## Background

Molecular parameters of key interest for monitoring the efficacy of anti-malaria interventions are those that quantify effects on transmission or incidence of infection and disease.

Vaccine trials provide regular follow-up samples of both comparator groups. In addition, the clinical episodes are detected and sampled. Provided the spacing of follow up surveys is in the range of 2-8 weeks, the force of infections (FOI) can be determined by genotyping all consecutive samples of all study participants. The molecular FOI is defined by new parasite clones appearing per time interval. Individual clones are identified using highly polymorphic molecular markers in conjunction with high resolution typing. _mol_FOI is easily detected despite a background of ongoing infections.

Simultaneously, blood samples from trial participants become available for RNA based detection and quantification of gametocytes by qRT-PCR. Vaccine effects on gametocyte prevalence can be detected by targeting gametocyte-specific transcripts.

## Materials and methods

We have evaluated RNA sampling techniques for malaria field surveys. Collecting samples directly into RNAprotect solution gave best results. Gametocytes were detected by qRT-PCR using marker pfs25.

In our cohort studies asexual parasites were genotyped using marker msp2 and fragment sizing by capillary electrophoresis [1,2]. In the vaccine trial (Phase 2b vaccine trial of Combination B conducted in Papua New Guinea [3]) a PCR-RFLP methodology was used. We have developed the statistical approaches to determining the actual number of *P. falciparum* clones aquired per time per individual host, corrected for imperfect detectability [4].

## Results

Molecular parameters describing the *P. falciparum* infection dynamics were estimated based on high precision genotyping data from cohort studies or from a clinical trial with repeated follow up bleeds at intervals between 2 weeks and 2 months. The full time-series of presence and absence of clones in consecutive samples from one individual forms the basis from which _mol_FOI, duration of infection, and clone detectability were estimated.

_mol_FOI in vaccinated children from the Combination B vaccine trial was significantly reduced in vaccine recipients only for parasites carrying a 3D7-type *msp2* allele corresponding to 3D7 MSP2 component of Combination B.

## Conclusions

We demonstrated proof of concept of this approach in a vaccine trial of the Combination B malaria vaccine. We propose to consider the _mol_FOI as an outcome measurement in vaccine trials and to collect and preserve in the field trial setting in parallel blood samples useful for RNA extractions for monitoring vaccine effects on transmission stages.
